# Attentional capture by alcohol-related stimuli may be activated involuntarily by top-down search goals

**DOI:** 10.1007/s00213-018-4906-8

**Published:** 2018-04-25

**Authors:** Chris R. H. Brown, Theodora Duka, Sophie Forster

**Affiliations:** School of Psychology, University of Sussex, Pevensey 1, Falmer, BN19QH UK

**Keywords:** Attentional bias, Reward, Alcohol, Incentive salience, Goal-driven attention, Top-down attention

## Abstract

Previous research has found that the attention of social drinkers is preferentially oriented towards alcohol-related stimuli (attentional capture). This is argued to play a role in escalating craving for alcohol that can result in hazardous drinking. According to incentive theories of drug addiction, the stimuli associated with the drug reward acquire learned incentive salience and grab attention. However, it is not clear whether the mechanism by which this bias is created is a voluntary or an automatic one, although some evidence suggests a stimulus-driven mechanism. Here, we test for the first time whether this attentional capture could reflect an involuntary consequence of a goal-driven mechanism. Across three experiments, participants were given search goals to detect either an alcoholic or a non-alcoholic object (target) in a stream of briefly presented objects unrelated to the target. Prior to the target, a task-irrelevant parafoveal distractor appeared. This could either be congruent or incongruent with the current search goal. Applying a meta-analysis, we combined the results across the three experiments and found consistent evidence of goal-driven attentional capture, whereby alcohol distractors impeded target detection when the search goal was for alcohol. By contrast, alcohol distractors did not interfere with target detection, whilst participants were searching for a non-alcoholic category. A separate experiment revealed that the goal-driven capture effect was not found when participants held alcohol features active in memory but did not intentionally search for them. These findings suggest a strong goal-driven account of attentional capture by alcohol cues in social drinkers.

**Electronic supplementary material:**

The online version of this article (10.1007/s00213-018-4906-8) contains supplementary material, which is available to authorized users.

Images of alcohol have been found to capture the attention of individuals who regularly consume alcohol (Field and Cox 2008, see Rooke et al. 2008 for meta-analysis). This attentional bias has been causally implicated in problem drinking: The bias correlates with craving for alcohol (Field et al. 2009), and training individuals to adopt the bias directly increases craving (Field and Eastwood 2005). This suggests that attentional bias towards alcohol cues may play a mediating role in the maintenance of hazardous drinking behaviour through elevating the craving for alcohol (Franken 2003, Field et al. 2016, although see Christiansen et al. 2015).

Within the general attention literature, it is established that the biasing of attention towards a particular stimulus can reflect either stimulus-driven mechanisms, resulting from the inherent attention-grabbing properties of the stimulus itself, or goal-driven mechanisms, resulting from the voluntary prioritisation of that class of stimulus (Corbetta and Shulman 2002). Understanding the underlying mechanism of alcohol bias has important implications for understanding models of addiction and for prevention and treatment of alcohol abuse.

A prominent theory of addiction, incentive sensitisation theory (IST), proposes that the attentional bias towards alcohol-related stimuli develops as a consequence of the repeated pairings between stimulus and the rewarding effects of alcohol (Robinson and Berridge 1993, 2001; Berridge and Robinson 2016). Through the repeated pairings with reward, the alcohol-related features take on a learned *incentive salience*, meaning that the features are now imbued with the ability to “grab” attention. Although the exact attentional mechanism is often left ambiguous, it is assumed that this bias occurs in a stimulus-driven manner. The incentive salient stimulus induces dopaminergic activity which directly influences selective attention, possibly independent of the intentions of the individual (Hickey and Peelen 2015). The current investigation, will, however, aim to test whether the attentional bias could alternatively be accounted for by a goal-driven attentional mechanism.

Evidence for the alcohol attentional bias comes from paradigms such as the dot-probe task, in which participants are instructed to respond to a dot in one of two locations, which are filled prior to the appearance of the dot by one alcohol image and one non-alcohol image (Townshend and Duka 2001). Heavy drinkers are typically slower to respond to the dot when it does not appear in the location that was previously occupied by the alcohol image, even when this image was presented only for 50 ms (e.g. Noël et al. 2006). This effect, among many others, occurs despite participants being instructed to ignore the alcohol image, which now acts as a distractor, and focus on detecting the target (e.g. Field et al. 2004).

It is important to note that the involuntary nature of the alcohol attention bias does not necessarily point to a stimulus-driven mechanism. In fact, over the past 26 years, evidence from the general attention literature has highlighted that involuntary attention should not always be assumed to reflect stimulus-driven mechanisms. Rather, paradoxical as it may seem, involuntary attention can actually be a direct consequence of voluntary top-down goals—a phenomenon known as “contingent capture” (cf. Folk et al. 1992). For instance, Folk et al. (2002) found that when participants were given a task goal to search for a specific colour in a stream of briefly presented stimuli (i.e. rapid serial visual presentation—RSVP), only irrelevant distractors which matched the search goal captured attention and interfered with target detection. Equally salient stimuli which did not match the current search goal did not interfere with target detection. Note that this goal-driven capture occurs despite participants being aware that the peripheral distractors were entirely task-irrelevant and despite the fact that attending to the distractors resulted in failure to detect the subsequent target. Hence, entirely involuntary attentional capture can result from a voluntary goal-driven attentional setting.

An involuntary yet goal-driven alcohol attentional bias could therefore plausibly occur among individuals who attentionally prioritise the detection of alcohol. Thus, the question is raised: Are social drinkers “on the lookout” for alcohol in their environment, with the result that they automatically notice it even when they are meant to be completing another task? Evidence suggests that heavy drinkers find viewing alcohol stimuli pleasant (Field et al. 2004). Regular social drinkers report enjoying thinking of alcohol and report that being a drinker is part of their explicit identity (Martino et al. 2017; Lindgren et al. 2013). Given that drinkers find alcohol pleasant to view and personally relevant, we argue that they may also be likely to adopt a voluntary goal to look out for it.

In terms of IST, the motivational effect of craving has also been found to influence voluntary goal-directed choice (e.g. Mackillop et al. 2010). There is also some evidence that dopaminergic activity is implicated in the voluntary maintenance of top-down goals, not just bottom-up automatic processing of stimuli (e.g. Frank et al. 2001). Thus, social drinkers who have learnt the incentive value of alcohol may be more motivated to search for alcohol features than non-drinkers, leading to involuntary contingent capture by alcohol stimuli.

A stimulus-driven account would predict that the alcohol attentional bias would be found regardless of the current attentional goals. It is notable, however, that investigations which have previously found evidence favouring the stimulus-driven account are limited to using paradigms, in which the task cannot be performed without some degree of intentional allocation of attention to the alcohol images. For instance, in previous tasks (e.g. the widely used dot-probe), the distractors are always presented in an attended location (i.e. the same location as the potential targets). To our knowledge, no evidence has suggested, nor has any theory of attention proposed, that it is possible to entirely ignore the features of a stimulus presented in an attended location. Thus, presenting alcohol images in a potential target location, which must be attended in order to perform the task, would make attentional processing unavoidable. Furthermore, it is notable that no actual cost is incurred by consistently attending to the alcohol images in the dot-probe. Because the images are predictive of the location of the target on 50% of the trials, attending to these images does not slow the overall reaction time. Favouring one set of images would give the same overall reaction time as if participants ignored those images, meaning that there is little incentive to try and ignore them. This raises the possibility that previous findings of the attentional bias for alcohol might be accounted for by social drinkers voluntarily attending to the alcohol images, given that they find these pleasant and personally relevant and there is no cost for doing so. In fact, when the target probe is consistently presented in a separate location from the alcohol images (e.g. 96% of trials), then attention can be effectively trained away from the alcohol cues (Schoenmakers et al. 2007). Thus, for a completely involuntary attentional bias to be measured, the alcohol images must appear in a distinct task-irrelevant location.

In the current investigation, we aim to establish whether the extent to which social drinkers adopt a top-down goal for alcohol can determine whether or not they exhibit an attentional bias towards completely task-irrelevant alcohol distractors. To test this, we adapted the RSVP paradigm used by Folk et al. (2002) to include alcohol images. Specifically, we instructed participants to search a stream of rapidly presented everyday objects for either alcohol, or a category of non-alcoholic stimuli, in different blocks. We presented alcohol and non-alcoholic distractor images in completely task-irrelevant parafoveal locations, which participants were instructed to ignore. Note that within this paradigm, it is possible not only to completely ignore the distractors but also attending to the distractors would result in the complete failure to detect the subsequent target. Therefore, participants are strongly motivated to avoid any voluntary allocation of attention to the alcohol distractors.

If a goal-driven mechanism can account for involuntary biases of attention in social drinkers, alcohol distractors should selectively disrupt task performance (target detection) when participants are currently searching for alcohol. Conversely, a stimulus-driven attentional bias, operating independent of the current goals of the individual, would result in a bias regardless of whether the participant currently holds an alcohol or a non-alcohol search goal.

## Experiments 1a, 1b, 1c

We conducted three versions of experiment 1 to test the replicability of our effect whilst adjusting for differences in task difficulty. Experiments 1a and 1b were identical, with the exception of the presentation speed which was slowed down from 83 (1a) to 100 ms (1b) in an attempt to equate task difficulty between the alcohol and non-alcoholic goals. Experiment 1c changed the non-alcohol stimulus category from pots/pans to shoes, for the same reason. Additionally, a larger sample was collected for experiment 1c in order to allow sensitivity to detect a potentially smaller stimulus-driven effect.

### Methods

#### Participants

Table 1 presents participant’s characteristics. The inclusion criteria required that participants must have consumed alcohol in the last month, were not currently abstaining, and were from the University of Sussex student subject pool. These participants were remunerated with either partial course credit or small cash payment. Informed consent was collected prior to participation, and ethics were approved by the University of Sussex Ethics Committee in accordance with the 1964 Declaration of Helsinki.

**Table 1:** The mean demographic and questionnaire data from across all four experiments and standard deviations are presented in brackets

	Sex	Age	Units (AUQ)	AUDIT	Positive arousal (AEAS)
Experiment 1a	7 females	22 (2.45)	21.43 (25.43)	8.0 (3.77)	7.19 (1.34)
	5 males				
Experiment 1b	13 females	20.44 (2.06)	12.68 (14.74)	11.94 (6.20)	7.48 (.95)
	3 males				
Experiment 1c	46 females	21.6 (3.91)	16.49 (11.13)	12.18 (6)	7.79 (1.09)
	14 males				
Experiment 2	24 females	21.37 (2.25)	18.91 (15.05)	13.21 (5.35)	7.71 (1.32)
	19 males				

Units of alcohol was measured by the Alcohol Use Questionnaire (AUQ; Mehrabian and Russell 1978) and reflects the number of units drank in a typical drinking week. The Alcohol Use Disorders Identification Test (AUDIT; Saunders et al. 1993) reflects not only the number of units drank per week but also the frequency of negative outcome from drinking alcohol. A score of 8 or above suggests a hazardous relationship with alcohol, the maximum score is 40. The positive arousal reflects the mean expectancy of a positive and high arousing outcome (e.g. feeling “lively”) immediately after consuming an acute dose of alcohol, recorded on a scale of 1 to 10. The score is a subscale taken from the Anticipated Effects of Alcohol Scale which reflects the reward stimulation from consuming alcohol (Morean et al. 2012)

Experiments 1a and 1b were intended to test whether a goal-driven attentional bias to alcohol could be induced; therefore, sample size calculations were conducted prior to data collection using Gpower software to determine which sample size would be suitable to detect a goal-driven effect (Faul et al. 2009). This revealed that to detect an effect size of *d* = .92 (two-tailed; *α* = .05; 1 − *β* = .80), a sample of 12 participants was required. However 13 participants were originally recruited due to one being excluded due to a programming error. The expected effect size for this power analysis was taken from a previous demonstration of goal-driven attentional bias to emotional faces (Brown et al. under review).^1^ The final sample size of Experiment 1b, after excluding one participant for currently abstaining from alcohol, was larger than 1a (*n* = 16) due to scheduling error.^2^

The intention of experiment 1c was to test whether a stimulus-driven attentional bias was evident in the current paradigm. We, therefore, increased the sample size to detect a smaller alcohol bias effect which has been found in previous studies. A power analysis revealed that a sample of 60 participants should be suitable to detect a small alcohol bias effect of *d* = .37 (two-tailed; *α* = .05; 1 − *β* = .8). This effect size was based on the 95% lower bound confidence interval of the meta-analytically computed relationship between alcohol consumption and an “implicit” cognitive bias towards alcohol, as reported by Rooke et al. (2008).

#### Alcohol Use Questionnaire (AUQ)

The AUQ is a 12-item questionnaire which measures the frequency and speed of the weekly consumption of specific alcoholic drinks, which allows the computation of the number of units drank per week and binge score (Mehrabian and Russell 1978).

#### Alcohol Use Disorder Identification Test (AUDIT)

The AUDIT is a 10-item scale which measures not only both the frequency and amount of alcohol consumed but also the negative behavioural consequences from alcohol, e.g. when drinking is concerning to others (Saunders et al. 1993).

#### Anticipated Effects of Alcohol Scale (AEAS)

The AEAS is a 22-item scale that measures the expected emotions immediately after consuming an imagined amount of alcohol (four drinks for females and five drinks for males). The scale is composed of four subscales varying along dimensions of arousal and valence (Morean et al. 2012). The main subscale of interest was the positive high arousal factor, as this factor will indicate whether individuals perceived alcohol to be rewarding (cf. Bradley et al. 2001).

#### Stimuli

**Fig. 1:**
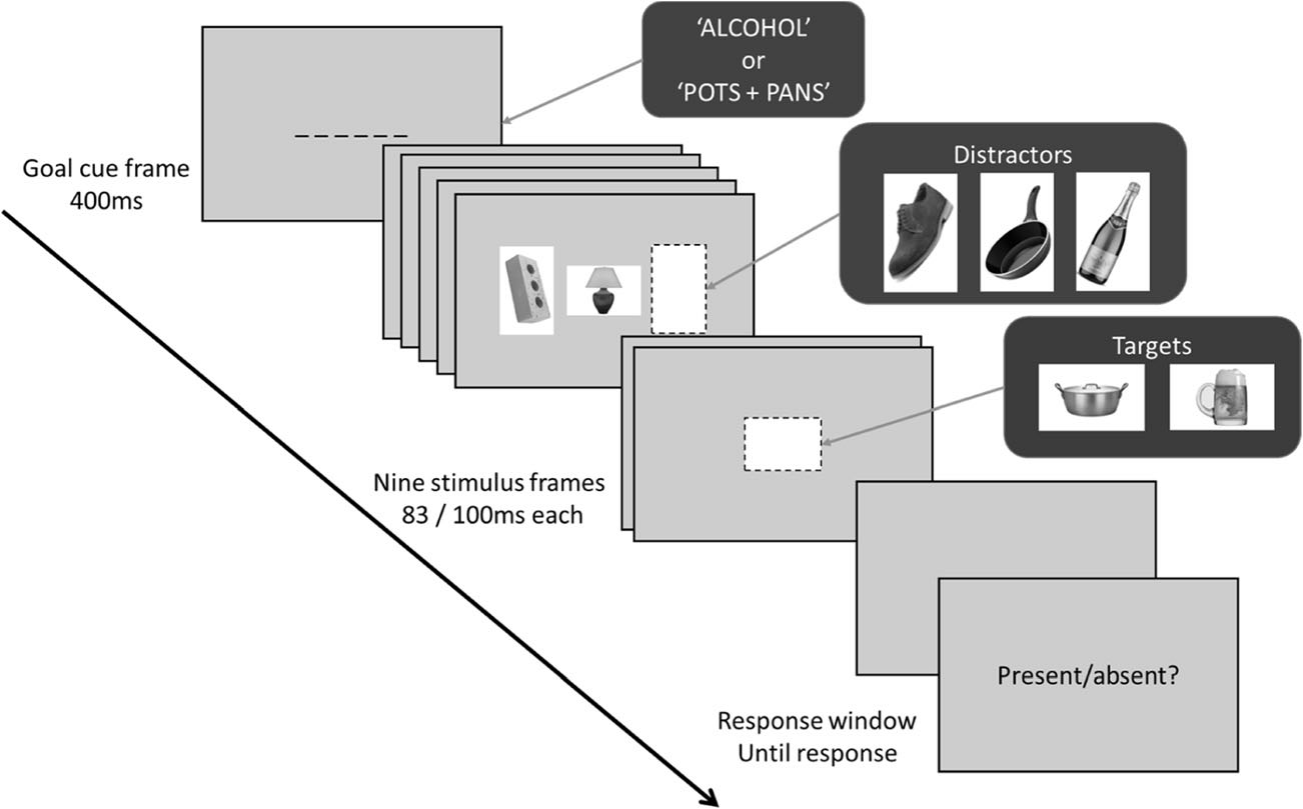
Structure of a single RSVP trial and stimuli used across the four experiments. At the start of each trial, participants were presented with a 400-ms goal cue prompt, with the target type for that block: alcohol or pots/pans (experiments 1a, 1b) or alcohol or shoes (experiment 1c). Each of the subsequent nine images in the RSVP appeared for 83 ms (experiment 1a) or 100 ms (experiments 1b, 1c, and 2) without interstimulus interval. In experiment 2, there was no prompt because they always had to detect cars in the RSVP stream; however, a pot/pan image or alcohol image was presented at the start of each trial for participants to retain in memory for the duration of the trial. At the end of each trial, participants identified whether a target had been present or absent. The irrelevant distractors were identical across all experiments, whilst the target type varied depending on what the search goal was (experiment 1a: pots/pans, alcohol; experiment 1b: pots/pans. Alcohol; experiment 1c: shoes, alcohol, experiment 2: cars)

Across all experiments, stimuli were presented using E-prime 2.0 software on a Dell 1707FP computer. The resolution was set to 1280 × 1024, and the viewing distance was maintained at 59 cm using a chin-rest. Example stimuli are presented in Fig. 1, and all stimuli are available online via the Open Science Framework (OSF: osf.io/9n8yq). All target and distractor stimuli were images of single objects on a plain white background. The images within each category were selected so that they formed a heterogenous visual category with multiple features, textures, and shapes. The alcohol stimuli were selected so that there were equal numbers of exemplars of spirits, wine, and beers—and half of these stimuli were presented in glasses, the other half in bottles. Pots/pans images were selected, so that there were a variety of materials and colours which formed the category (e.g. ceramic, steel, and copper). Approximately half the exemplars were frying pans, the other half pots. The shoes were selected so that there were multiple different types of shoe (e.g. sports trainers, high heels, boots, and men’s formal shoes). Men’s shoes and women’s shoes were presented approximately equally, though there were some unisex shoes presented. These image selection criteria thus encouraged participants to form a search goal for a general category of objects, rather than any single feature.

The angles which the shoe and alcohol images appeared were more uniform than the pots/pans; we therefore rotated several exemplars from these categories, so that these categories were matched on the variability of stimulus orientation. The alcohol target category contained 12 full colour images of different types of alcohol. In experiments 1a and 1b, the non-alcohol target category contained 12 images of different types of pots/pans. In experiment 1c, the non-alcohol target category contained 12 images of shoes.

Three categories of distractor images were presented in each experiment: alcohol, pots/pans, and shoes. In experiments 1a and 1b, the shoe category was included as a completely goal-incongruent category (i.e. not matching either task search goal), whilst in experiment 1c the pots/pans were the goal-incongruent category. Each distractor category was composed of 16 images, which were visually similar to the target images of the same category but were never the same exemplars. All distractor and target images appeared an equal number of times within each condition. The distractors appeared to the left and right of the central stream with a gap of .5° between them. All centrally presented distractors measured 3.44° × 2.29°, whilst the parafoveal distractors measured 2.98° × 4.58°.

In total, 408 non-alcoholic filler images were selected to appear in the central stream. These were composed of 24 different everyday household objects with 17 different exemplars of each of these objects (see Appendix Table 3 for full list of non-alcoholic items stimuli). An additional 48 non-alcoholic object images were selected to appear as fillers in the parafoveal locations, these were composed of the same 24 object categories with two exemplars from each category. The parafoveal filler served to fill the other distractor location not occupied with an alcohol, shoe, or pot/pan distractor. All stimuli were sourced from Google images and appeared in isolation from other objects on a white background. During the task, these images were presented on a grey coloured screen (red/green/blue balance: 192, 192, 192). All images appeared four times across the experiment. Due to potential similarity to the shoe targets, in experiment 1c socks were removed from the filler stimuli and were replaced with 19 lamp images; 17 in the central set, two in the parafoveal set.

#### RSVP task

In experiment 1a, participants were instructed to search in a central RSVP stream of nine images for an object from a specific category, each image appeared for 83 ms. The task consisted of two blocks of 96 trials; in one block participants were instructed to search for “ALCOHOL”, in the other “POTS + PANS,” and this search order was counterbalanced between participants. Participants received 400-ms reminders of what the search goal was before each trial, i.e. “alcohol” or “pots and pans.” At the end of each trial, participants had to report whether they believed the target had been present or absent. Responses were made using the “c” and “m” keys, with the key-response assignment counterbalanced between participants. On half of the trials the target was present; the other half it was absent. The response screen contained only the words “present/absent?” and disappeared once the participants had responded.

When present, the target image could appear at positions five, six, seven, or eight in the RSVP stream. When absent that particular position in the stream was filled with a filler image. Distractor images appeared to the left and right of the central stream, one position was filled with either a shoe, pot/pan, or an alcohol distractor, whilst the other position was occupied with a filler image of the same size. Shoe, pot/pan, and alcohol distractors each appeared on a third of the trials in each block. These distractors always appeared two images prior to the target (i.e. lag 2). All within participants’ variables were counterbalanced within each block. Before the task started participants completed a 16-trial practice block of equal alcohol and pot/pans targets. Participants were verbally instructed before the main task that the target category would only vary between blocks, not between trials, and that the participants should ignore every image outside of the central stream.

Changes were made to experiment 1b due to the pot/pan targets being more difficult to detect than the alcohol targets in experiment 1a. We, therefore, slowed the stimulus presentation time down to 100 ms per image. This is more in line with previous RSVP tasks which have found implicit attentional capture by affective stimuli (Most et al. 2005). Despite the slower presentation time in experiment 1b, pot/pan targets were still detected less accurately than alcohol targets; therefore, we switched the non-alcoholic targets in experiment 1c to salient shoe images. The trials now started with an instruction to search for “SHOES” instead of “POTS + PANS.” The prompt in the response screen was also changed from “present/absent?” to a single “?” to avoid any influence of word order on responding.

#### Procedure

For experiments 1a and 1b, participants were tested in a dimly lit testing room at the University of Sussex. After providing informed consent, participants were given task instructions and then completed the practice block with supervision from the experimenter, after which they completed the RSVP task on their own. Participants then completed pen and paper versions of the AUDIT, AUQ, and AEAS in a random order. The experiment took approximately 25 min to complete. In experiment 1c, the procedure was identical to experiments 1a and 1b, with the exception that the questionnaires were presented using Inquisit 5 in order to automate randomisation of the questionnaire order. Half the participants completed the questionnaire prior to the RSVP task and half afterwards. Finally, participants were debriefed as to the full aims of the study.

#### Analytic strategy

Across experiments 1a, 1b, and 1c, we conducted the same analyses. The dependent variable used was A-prime (*A′*) detection sensitivity index which controls for response bias; this was computed based on the proportion of hits and false alarms made during the present/absent task response (Stanislaw and Todorov 1999; Zhang and Mueller 2005). *A*′ ranges from .5, which indicates that a signal cannot be distinguished from noise (i.e. chance detection), to 1, which corresponds to perfect detection of the target. In order to determine whether there was any significant difference in *A′* across conditions, each individual study was initially analysed using a 2 × 3 repeated measures ANOVA in SPSS statistical software, using current goal type (alcohol/non-alcohol) and distractor type (alcohol/goal congruent non-alcohol/irrelevant non-alcohol) as the factors.

To follow up these comparisons and to determine the overall strength of the effect, we conducted pairwise comparisons across three studies using an internal meta-analysis. Four pairwise comparisons were computed; these were between the goal congruent distractors and the irrelevant distractor, in both search goal conditions (individual experiment comparisons are reported in Online Resource 1). The meta-analysis was conducted using the Metafor statistical package in R which weighted each experiment by its sample size (as described in Aloe and Becker 2012, Viechtbauer 2010). In all experiments, *A′* scores were significantly skewed; therefore, a DerSimonian-Laird random effects model was used to compute the cumulative effects and confidence intervals, which is robust to violations of normality and is suitable for calculating cumulative effects from a small number of studies (DerSimonian and Laird 1986; Kontopantelis and Reeves 2012).

Bayes factors were calculated for all pairwise comparisons across experiments, as well as the cumulative effect. A Bayes factor compares evidence for the *experimental hypothesis* (positive attentional capture by alcohol versus an irrelevant distractor) and the *null hypothesis* (zero capture by alcohol versus an irrelevant distractor). The Bayes factor ranges from 0 to infinity. The strength of this evidence is indicated by the magnitude of the Bayes factor; values greater than three or less than .33 indicate substantial evidence for either the experimental or null hypothesis, respectively. A value closer to 1 suggests that the result is insensitive and any difference is “anecdotal” (Dienes 2008, 2011, 2014, 2016).

The Bayes factors were computed using a modified version of Baguley and Kaye’s (2010) R code (retrieved from Dienes 2008). To compute the factor, I used a half-normal distribution with a mean of zero to reflect the null hypothesis. The standard deviation of the distribution for all pairwise comparisons was set to .10, which is the plausible raw effect size for a difference between goal-congruent distractor and irrelevant distractor.^3^ For meta-Bayes factors, used for the overall population mean, the effect was computed sequentially using Zoltan Dienes online calculator; first, combining the raw effect sizes and standard error of experiments 1a and 1b, then combining this cumulative posterior value with the mean and standard error of experiment 1c (Dienes 2008; Rouder and Morey 2011).

## Results

An initial review of the participants’ self-reported drinking-related scores from experiments 1a, 1b, 1c, and 2 revealed that they were within the range of previous investigations, which found attentional biases towards alcohol cues (Tibboel et al. 2010, Ramirez et al. 2015, Sharma et al. 2001, DePalma et al. 2017, see Table 1). Additionally, we note that the samples contained a large number of participants who would likely attribute incentive value to the alcohol stimuli: 98% of participants reported expecting some degree of positive arousing outcome from consuming alcohol (scored > 5; Morean et al. 2012); 78% were classed as problem drinkers by the AUDIT and therefore at risk of substance dependence (scored > 8; Saunders et al. 1993), and 52% were classified as binge drinkers on the AUQ (scored > 24; Townshend and Duka 2005). Exploratory analyses using participant characteristics are reported below, with full details presented in Online Resource 2.

**Fig. 2:**
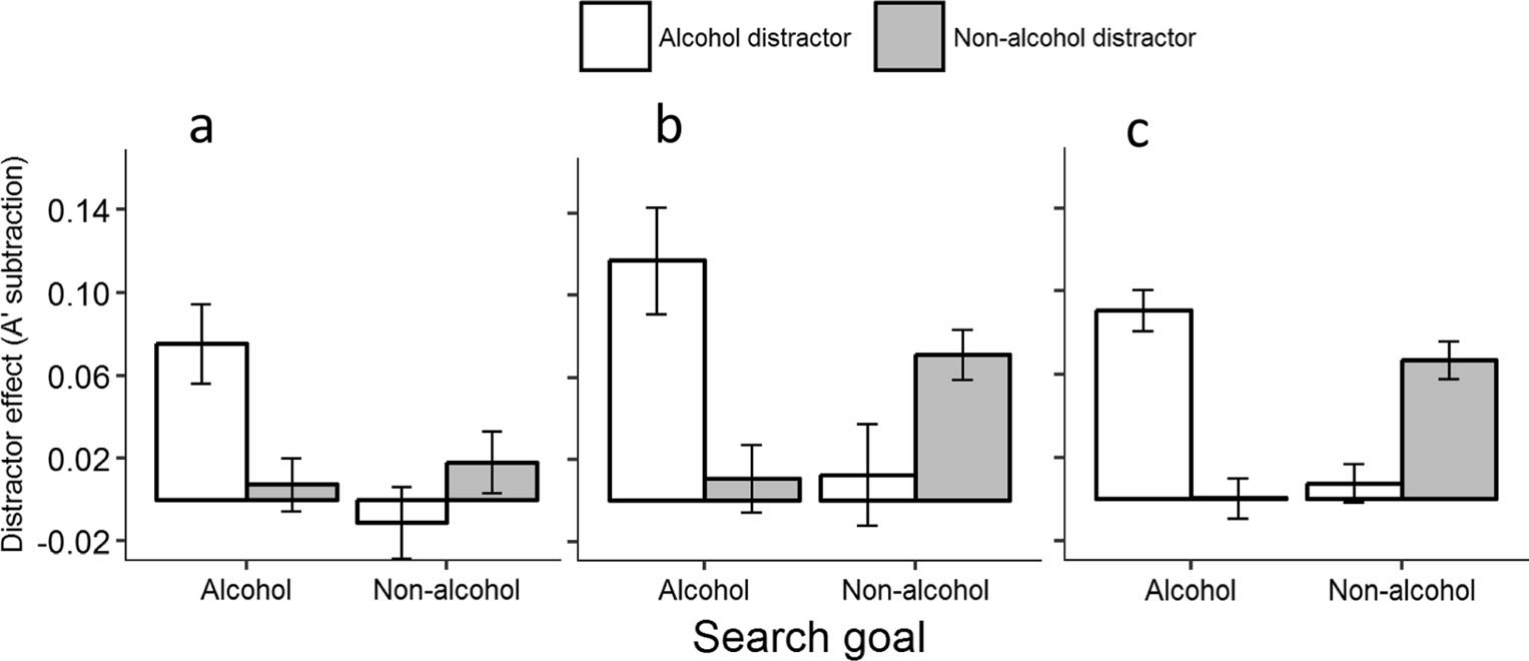
Graph depicting the mean distractor effects across experiments 1a, 1b, and 1c. The distractor effect reflects the subtraction of the *A′* detection sensitivity score when the distractor was of the same category as one of the search goals, from the distractor which is never searched for. This distractor effect was calculated for both search goal conditions. Error bars reflect within-participants’ standard error

Mean *A′* and standard deviations from each condition across all experiments are presented in Table 2 and see Fig. 2 for the distractor effects, which show the subtraction of *A′* scores when the distractor is goal relevant from the distractor which is never congruent with the search goal. Experiments 1a and 1b both showed significant effects of search goal, *p*’s < .007, thus revealing that the pot target was harder to detect than the alcohol target (experiment 1a: alcohol *M* = .81, *SD* = .07 vs pots/pans *M* = .73, *SD* = .1; *F*(1,11) = 17.42, *p* = .002; experiment 1b: alcohol *M* = .80, *SD* = .10 vs pots/pans *M* = .73, *SD* = .15; *F*(1,15) = 9.76, *p* = .007). The effect of search goal was however non-significant for experiment 1c, confirming that our adjustments to the task were successful in equating the accuracy level for detection of shoes versus alcohol targets, *M* = .80, *SD* = .09 vs *M* = .80 *SD* = .09; *F*(1,59) = 1.34, *p* = .252.

**Table 2:** The mean *A*′ scores and standard deviations from across all conditions in the four experiments

	Search goal	Distractor type		
		Alcohol	Pots	Shoes
Experiment 1a (*n* = 12)	Alcohol	.76 (.10)	.83 (.04)	.84 (.05)
	Pots/pans	.74 (.10)	.71 (.11)	.73 (.10)
Experiment 1b (*n* = 16)	Alcohol	.73 (.14)	.83 (.05)	.84 (.03)
	Pots/pans	.74 (.16)	.68 (.16)	.75 (.11)
Experiment 1c (*n* = 60)	Alcohol	.74 (.14)	.83 (.04)	.83 (.04)
	Shoes	.82 (.06)	.82 (.05)	.76 (.13)
Experiment 2 (*n* = 43)	Alcohol	.82 (.07)	.82 (.07)	.82 (.08)
	Pots/pans	.83 (.06)	81 (.08)	.83 (.07)

*A*′ was computed from the frequency of hits and false alarms made during the present/absent judgement. *A′* is a detection sensitivity index which ranges from .50 to 1, with .50 reflecting chance detection and 1 reflecting perfect detection of the target

Regardless of any main effect of search goal, the pattern of results concerning the distractors was identical across all three experiments. In each, the distractor effect was significant, showing that some distractors had reduced detection sensitivity of the targets (experiment 1a: *F*(2,22) = 5.22, *p* = .014; experiment 1b:, *F*(2,30) = 11.09, *p* = .001 (Huynh-Feldt corrected); experiment 1c: *F*(1,118) = 26.59, *p* < .001). Critically, all three experiments revealed the main effect of distractor to be qualified by a significant interaction between search goal and distractor type, thus suggesting that some distractors interfered more with the task when participants were searching for a congruent target (experiment 1a: *F*(2,22) = 5.79, *p* = .019; experiment 1b: *F*(2,30) = 12.47, *p* = .001 (Huynh-Feldt corrected); experiment 1c: *F*(1,118) = 25.12, *p* < .001). Specifically, as can be seen in Fig. 2 and as predicted by a goal-driven account of alcohol-related attentional biases, distractor interference was observed only during search conditions that involved a goal for that distractor type. To further delineate these distractor effects and their interactions with search goal, we computed pairwise comparisons between distractors when they were both goal congruent and goal incongruent, meta-analytically (see Fig. 2; see Online Resources 1 for individual experiment analyses).

### Internal meta-analysis

**Fig. 3:**
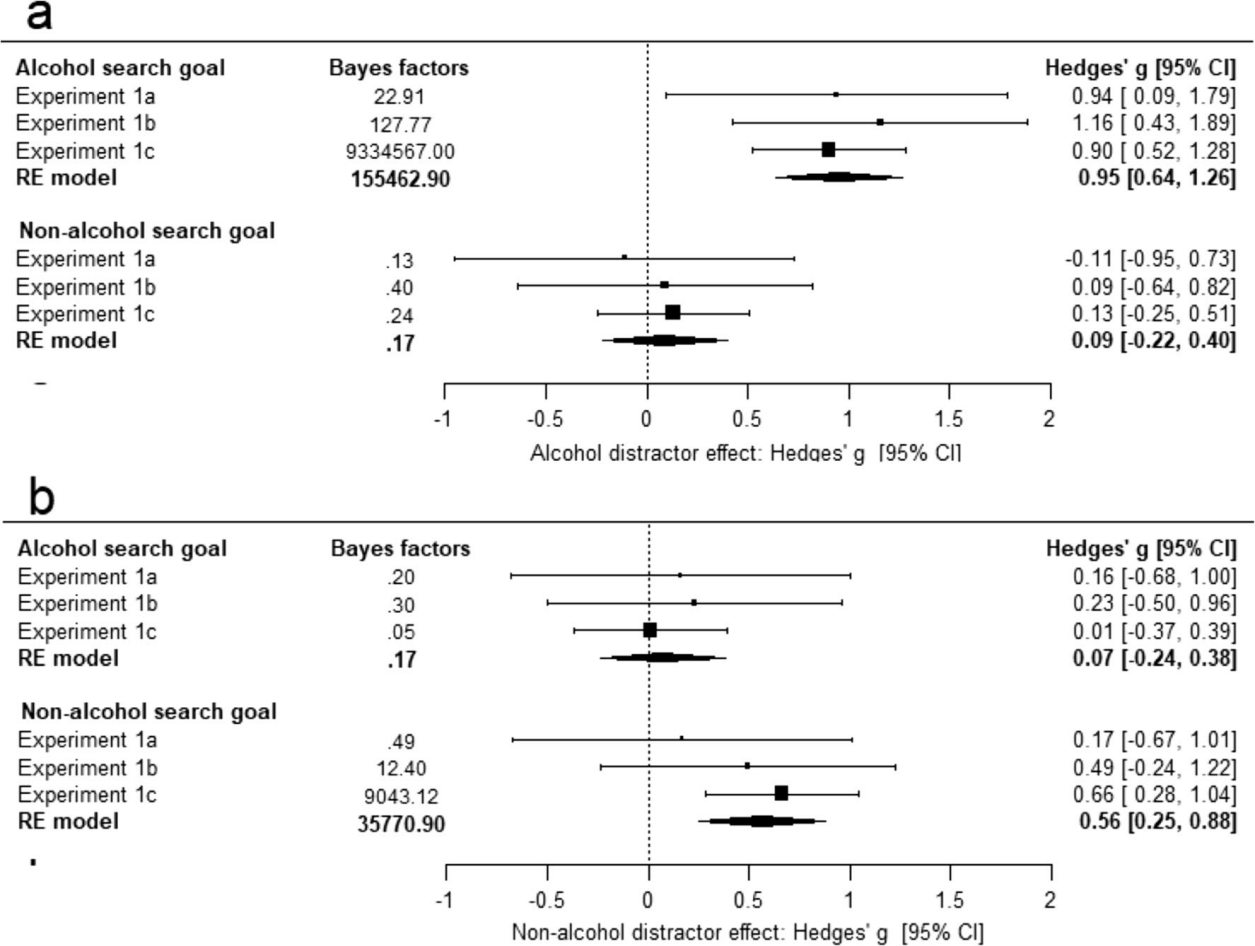
Forest plots presenting the random effect model of the cumulative Hedges’ *g* effect sizes, confidence intervals, and Bayes factors. Values for each individual study are also presented. **a** Reflects the distractor effect for the goal congruent alcohol distractor versus a completely irrelevant non-alcoholic distractor, when searching for alcohol (top; goal-driven effect) and when searching for a non-alcoholic object category (bottom; stimulus-driven effect). **b** Reflects the distractor effect for a goal congruent non-alcohol distractor versus a completely irrelevant non-alcohol distractor, whilst searching for alcohol (top; stimulus-driven effect) a non-alcoholic object category (bottom; goal-driven effect)

See Fig. 3 for the meta-analytically computed effect sizes and confidence intervals, as well as Bayes factors. As hypothesised, when comparing the alcohol distractor effect versus the completely task-irrelevant distractor, there was a consistent and large effect size (Hedges’ *g* = .95) across all three experiments, with Bayes factors also showing very strong evidence in favour of the experimental hypothesis. We note that the large alcohol goal-driven effect was similar across experiments, regardless of sample size, suggesting that the goal-driven effect was consistent and replicable (cf. Button et al. 2013).

Similarly, when the non-alcohol distractor was congruent with the contents of the current non-alcohol search goal, there was a medium sized decrement (Hedges’ *g* = *.*56) in detection sensitivity versus the completely task-irrelevant non-alcohol distractor. The Bayes factors revealed that overall, there was strong evidence favouring the experimental hypothesis, although this was not true across all experiments, with evidence favouring the null in experiment 1a.

Interestingly, the goal-driven alcohol distraction was larger than the non-alcoholic goal congruent distraction: experiment 1a: *t*(11) = 2.44, *p* = .031; experiment 1b: *t*(15) = 1.96, *p* = .068; experiment 1c: *t*(59) = 2.97, *p* = .004. There are multiple potential causes for this difference, though it could hint at an interaction between the qualities of stimulus features and participants’ current goals (see “General discussion”).

In contrast to the large and consistent goal-driven distractor effect when the alcohol distractor was incongruent with the current search goal, there was a non-significant and negligible effect size, when comparing it to the goal-incongruent non-alcohol distractor (Hedges’ *g* = .09). Overall, the Bayes factors showed evidence for the null hypothesis (Bayes factor < .33). When the non-alcohol distractor was incongruent with the current search goal, there was also a negligible and non-significant effect size, when compared to the completely task-irrelevant distractor (Hedges’ *g* = .07). The Bayes factor also showed evidence favouring the null hypothesis (Bayes factor < .33). The evidence, therefore, suggests that a distractor only resulted in interference when it was congruent with the current search goal, regardless of whether it was alcohol or a neutral category. The same distractors which capture attention under these conditions had no effect upon performance when they were incongruent with the current search goal. This was true for both non-alcohol stimuli and alcohol stimuli.

### Effects of degree of alcohol dependence

To explore whether the current alcohol dependence may have influenced the current findings, we divided participants from experiment 1c into low and high alcohol dependence risk groups (both *n* = 14) based on their AUDIT score (low < 8; high > 15; Babor et al. 2001). Including this two-level factor in the original 2 × 3 ANOVA revealed no significant interactions with task performance, all *p*’s > .743, *ƞ*^2^_p_ < .01. On the other hand, Bayesian pairwise comparisons between alcohol and task-irrelevant distractors for each group revealed that high-risk drinkers showed some evidence favouring a stimulus-driven effect, *p* = .033, *B*_H[0, .10]_ = 2.39, whilst the low-risk drinkers showed evidence favouring the null hypothesis of no stimulus-driven effect, *p* = .750, *B*_H[0, .10]_ = .17. Thus, it provides some support for IST’s proposal of a goal-independent attentional bias, though we note that the significant effect would not have survived Bonferroni corrections for multiple comparisons (*α* = .013), and the Bayes factor showed only weak evidence (Bayes factor < 3). Both groups showed evidence of goal-driven bias to alcohol, *p*’s < .009, *B*_H[0, .10]_ > 18.52.

Further exploratory correlation analyses using goal-driven and stimulus-driven alcohol distractor effects in experiment 1c and all alcohol relevant measures (AUDIT, AEAS positive arousal, units drank per week, AUQ binge score) revealed no significant correlations, all *r* < .16, *p* > .213. See Online Resource 2 for further details of these analyses.

## Experiment 2

The internal meta-analysis across our first experiment suggests that attentional capture by alcohol stimuli in the current task can be accounted for by a goal-driven mechanism. Experiment 2 sought to further clarify the precise mechanism underlying these effects. Note that our manipulation of goal-driven attention in experiment 1 is also likely to have manipulated the contents of visual working memory (VWM), in that participants may have maintained a representation of their search target throughout the search. Previous research suggests that merely holding information in VWM can bias attention (for review see Soto et al. 2008). For example, when participants were instructed to hold an image of palatable food active in VWM, task-irrelevant food images which matched this representation captured attention during a concurrent visual task (Higgs et al. 2012; Kumar et al. 2016). As such, it was important to consider whether the results of experiment 1 might reflect the role of more passive top-down VWM maintenance rather than resulting from a deliberate top-down attentional goal. To address this, experiment 2 modified our original paradigm, so that the contents of VWM were manipulated, whilst the primary search goal remained constant. Participants performed the RSVP task searching for an alcohol irrelevant category (cars), whilst also maintaining either alcohol-related or alcohol-unrelated (pots/pans) stimuli in VWM as part of a separate memory task. If VWM maintenance alone can explain the findings of experiment 1, similar results would be expected in this new experiment.

## Methods

### Participants

Forty-eight participants were initially recruited, though five were excluded from the analysis due to performing at chance on either the pots/pans or alcohol condition of the memory task. Sample size was based on the maximum number of participants that could be recruited over a 2-month period (see participant details in Table 1). This maximum time period stopping rule was chosen to collect the largest possible sample because we had no knowledge of what effect to expect, a priori. To confirm that the sample collected was adequately powered, we conducted a post hoc power analysis using the effect size from the interaction term of the repeated measures ANOVA (*ƞ*^2^_p_ = .015; see below). This revealed that there was adequate power to detect this effect within experiment 2, (1 − *β* = .80; Faul et al. 2009, Cohen 1988).

### Stimuli and procedure

The task and stimuli were identical to experiment 1b, with the following exceptions. At the start of each trial, a 1000-ms fixation cross was presented, which was followed by a 500-ms memory cue, measuring 5.14° × 3.35°, which participants were instructed to hold in memory throughout the RSVP search task. This was followed by a 400-ms ISI that preceded the RSVP stream. The RSVP task was similar to previous studies, except that the search target was a car (selected from one of 24 different car images). After the participant had responded to the present/absent judgement, a memory probe was presented from the same category as the memory cue. Participants had to judge whether the memory probe was the same or different from the memory cue they held in memory, they responded with “s” for same and “d” for different. On half the trials, the cue and probe matched. After this second response, participants were presented with feedback for the memory task, which appeared for 600 ms. Trials were separated with 100 ms of a white noise image filling the screen. All within participants’ variables were counterbalanced within each block; there were two blocks which were made up of 96 trials.

In one block, the memory cue was one of 24 alcohol images; on the other block, the memory cue was one of 24 pots/pans images. Each image consisted of different alcohol types or different pots/pans in a single scene.^4^ All additional images in this task were sourced from Google images. The order of these blocks was counterbalanced between participants. At the beginning of the task, participants were given a 16-trial practice block without any distractors. Half the participants completed the questionnaires prior to the task, half after.

## Results

In order to ensure that a VWM representation was active in the trials analysed, we removed all trials (10%) where participants incorrectly reported whether the probe was same/different from the cue. Rerunning the analyses with all trials included did not change the pattern or significance of the results. The RSVP target detection sensitivity (*A′*) was entered as the dependent variable in a 2 × 3 ANOVA, with active memory type (pots/pans, alcohol) and distractor (pots/pans, alcohol, shoes) as factors. For means and standard deviations see Table 2. The main effect of memory contents was non-significant, *F*(1,42) = .36, *p* = .550, *ƞ*^2^_p_ = .01, as was the main effect of distractor type, *F*(1,84) = 1.17, *p* = .316, *ƞ*^2^_p_ = .03. Importantly for our hypothesis, the interaction between memory contents and distractor type was non-significant, *F*(2,84) = .64, *p* = .529, *ƞ*^2^_p_ = .02, thus suggesting that there was no difference between the distractor type when it was congruent with the contents of VWM compared to when it was incongruent. To further test the sensitivity of this analysis, we conducted Bayesian pairwise comparisons. The data were significantly skewed meaning that follow-up analyses were supplemented with bootstrapped confidence intervals which are robust to violations of normality (Field 2013).

Follow-up Bayesian comparisons revealed no evidence of interference from alcohol (vs shoe) distractors, regardless of whether VWM contained alcohol images, *t*(42) = .21, *p* = .838, 95% CI [− .02, .2], *B*_H[0, .10]_ = .1, or pots and pans, *t*(42) = .04, *p* = 859, 95% CI [− .02, .02], *B*_H[0, .10]_ = .11. Note that this result meets the < .33 criteria for a sensitive null result (Dienes 2008). It therefore appears that despite the alcohol imagery being active in working memory, there was no biasing effect towards visually similar alcohol distractors. There was also no evidence of interference from pot (versus shoe) distractors either during the alcohol VWM condition, *t*(42) = .18, *p* = .859, 95% CI [− .01, .01], *B*_H[0, .10]_ = .09, or the pot VWM condition, *t*(42) = 1.71, *p* = .094, 95% CI [> − .01, .03], *B*_H[0, .10]_ = .62.

## General discussion

Across three experiments, the findings demonstrated that when participants held a search goal for alcohol-related targets, there was a consistent attentional bias to alcohol distractors. This occurred at presentations as brief as 83 ms and when the distractors were completely task-irrelevant, thus suggesting that an early and involuntary bias was induced by the search goal. Furthermore, Bayesian analyses revealed that this bias was absent when participants were searching for a non-alcoholic category of objects. Additionally, a null effect was found when participants held the alcohol features in VWM, but did not prioritise them as a search goal. Taken together, these results provide a clear demonstration that an involuntary attentional bias towards alcohol stimuli can be induced by the deliberate prioritisation of alcohol as a top-down search goal.

Our results are inconsistent with a stimulus-driven effect independent of the current search goal, as predicted by IST (Berridge and Robinson 2016). Although the present series of experiments cannot rule out the possibility that purely stimulus-driven effects might be observed in certain contexts, the present data suggest that a seemingly stimulus-driven effect may in fact be dependent on search goals driven by the individual’s desire to consume alcohol. We note that alcohol biases have exclusively been found among a group of individuals (i.e. drinkers) known to find alcohol imagery to be pleasant and personally relevant, who might hence reasonably choose to attend to these images (Field et al. 2004, Lindgren et al. 2013). Furthermore, previous evidence for the alcohol bias is derived from tasks such as the dot-probe, in which not only is there little motivation to follow the instruction to ignore the alcohol (in that there is no performance cost to doing so) but also in which the task instructions necessitate the allocation of attention to the location of the images, effectively making them impossible to completely ignore. Taken together with our demonstration that the bias can be induced by manipulating goal-driven mechanisms, it appears that the stimulus-driven account should be questioned.

A goal-driven account of attentional bias to alcohol stimuli could also explain some previous inconsistencies in the literature. Although overall attentional biases are found towards alcohol (Field and Cox 2008), more recently the attentional bias towards alcohol has been found to fluctuate over the duration of a dot-probe task (Gladwin 2017). Such a fluctuation effect could potentially be explained by the ebb and flow of goal priority, as individuals may switch between searching for alcohol cues and following the instruction to detect the dot-probe, which does not require much attentional engagement.

Integrating the current results into IST, it appears that the incentive value may not directly guide involuntary attention to reward-associated features. Rather, it may be that the incentive associations of a stimulus increase the likelihood that that object will be voluntarily searched for. This search goal could then induce an involuntary bias to the reward-associated features across the visual field. Indeed, it would make sense that a person who values alcohol would be likely to intentionally search for alcohol in their environment more than a less valued stimulus. An interesting feature of our results is that whilst our manipulation of search goal induced capture by alcohol and non-alcohol stimuli alike, the alcohol attentional bias was consistently stronger than the non-alcoholic goal-driven effect. This finding cannot reflect a purely stimulus-driven effect, because there was no evidence of distraction by the same stimuli when they were incongruent with the search goal. It may still indicate that high incentive salience of the stimuli interacts with the search goal, amplifying the goal-driven effect. Alternatively, perhaps the attentional capture was goal-driven, but the level of disruption was magnified due to craving induced by the alcohol stimuli.

One limitation of the current investigation is that our sample (by design) did not include sufficient variation in alcohol dependence to fully explore the relationship between levels of dependence and attentional capture. For now, we note that our follow-up analyses (outlined in Online Resource 2) found evidence favouring a very small alcohol distraction effect in the stimulus-driven condition of experiment 1c in high alcohol-dependent individuals, as measured by the AUDIT (Babor et al. 2001). We do not, therefore, discount the possibility that a stimulus-driven capture may occur for some individuals, as predicted by IST, though we note that any goal-driven effect is measurably greater than the stimulus-driven effect for these same individuals. We also note that there was only weak evidence favouring this stimulus-driven effect, which would not have survived Bonferroni correction. Future work should, therefore, aim to replicate the same findings in a larger sample of individuals who are currently categorised as alcohol-dependent or who currently crave alcohol. Furthermore, it remains possible that stimulus-driven capture could be observed more strongly in individuals with more severe alcohol addiction, such as those receiving in-patient treatment.

The term “goal-driven attention” is often discussed primarily in terms of the voluntary direction of attention in line with the task instructions (e.g. Theeuwes 2010). Our results, however, highlight that goal-driven attention is more complex and should not be conflated with voluntary attention. As we have demonstrated, a voluntary attentional goal can have involuntary attentional consequences, when participants searched for alcohol in one location they could not ignore alcohol in an irrelevant location, despite clear instructions to do so and despite an obvious performance cost to attending to the irrelevant alcohol. It therefore appears that there is a distinction between declarative task rules and goal-driven attention, which is often ignored in models of attention and addiction. In relation to alcohol, a heavy drinker may declare that they want to reduce their intake of alcohol when visiting the doctors, but they would likely exhibit different behaviour when in a bar where alcohol is present and the incentive value more apparent, leading them to prioritise the goal to search for alcohol in their environment.

In the current investigation, we found attentional capture only when the alcohol image was the primary search goal, but not when it was held in VWM. This finding appears to somewhat conflict with previous evidence that holding imagery in VWM can involuntarily bias external attention (e.g. Kumar et al. 2016). One reason for this could be that the current task required participants to search for a complex category of images in a perceptually demanding RSVP task (cf. Lavie 2005). It has recently been found that a secondary stimulus active in VWM only biases attention when the primary task is simple, such as when the target is a simple shape repeated across trials (Gunseli et al. 2016). What this does reveal is that alcohol cues are not automatically prioritised in attention and if an individual’s attention is sufficiently engaged with a competing goal this individual would not orient attention to congruent alcohol cues, despite those being active in memory.

In terms of applications, our results suggest that the attentional bias to alcohol was eliminated for many individuals when they were searching for non-alcoholic objects, even when they held an alcohol image in memory. This therefore suggests that interventions which encourage problem drinkers to pursue a competing attentional goal could be effective in disrupting attentional bias to alcohol and hence preventing this bias from leading to the escalation of craving (Field 2005, Franken 2003). This idea is consistent with evidence that individuals who were more satisfied with their non-alcohol related life goals were less prone to hazardous drinking, when compared to those who found their non-alcohol related goals unsatisfying (Cox et al. 2002). Further, the absence of a stimulus-driven distraction by alcohol in many participants suggests that attentional bias retraining might be improved by training participants to search for a single competing pleasant category (i.e. training participants to search for smiling faces in the presence of alcohol cues), rather than attempting to train avoidance of alcohol (i.e. training participants to search for a target away from an alcohol image and towards random non-alcoholic objects; Schoenmakers et al. 2007).

In summary, we have demonstrated that a consistent involuntary attentional bias to alcohol in social drinkers can be induced or blocked through a goal-driven mechanism. The present study is not definitive evidence of a goal-driven mechanism as the *only* driver of involuntary attention to alcohol cues; however, our clear demonstration of goal-driven alcohol attentional bias raises the possibility that effects previously assumed to be stimulus-driven could, actually, occur as an unintended outcome of voluntary top-down processes.

### Electronic supplementary material

ESM 1 (DOCX 15 kb)

ESM 2 (DOCX 21.6 kb)

## Appendix

**Table 3:** A list of all the categories of non-alcoholic filler stimuli across the four experiments. Twenty-four different categories were presented in each experiment; each category consisted of 19 exemplars, with two presented in parafoveal positions and 17 presented in the central RSVP stream. The 19 sock stimuli presented in experiments 1a, 1b, and 2 were replaced by 19 lamp stimuli in experiment 1c due to the sock stimuli’s relation to the shoe targets. All images can be found on the Open Science Framework (osf.io/9n8yq)

Non-alcohol filler categories in experiments 1a, 1b, 1c, and 2	
Bags	
Bed	
Belts	
Books	
Boxes	
Brushes	
Cleaning brushes	
Computer accessories	
Cupboards	
Cushions	
Cutlery	
Desks	
Electrical fans	
Gloves	
Pens	
Printers	
Rocks and bricks	
Sheds	
Socks	
Sofas	
Suitcases	
Towels	
Washing machines and dishwashers	
Wood	
“Lamps” replaced “socks” in experiment 1c	

## Footnotes

1 Note that this effect size is also in line with, or smaller than, published demonstrations of goal-driven capture using the RSVP task with irrelevant distractors. Wyble et al. (2013) found an average effect of *d* = 1.38 (*SD* = .57) across four experiments, and Folk et al. (2002) found an average effect of *d* = 1.50 (*SD* = .31) across three experiments.

2 We did not reject these participants in case further exclusions were required during analysis. Excluding the additional four participants did not alter the significance of the results—all key findings would have reached significance at *p* < .05 had we stopped after 12 participants.

3 The prior was based on a previous investigation in our lab which investigated capture effects by emotional faces in an identical RSVP task (Brown et al. under review). Experiment 1a and 1b’s sample sizes were *n* < 30, therefore, following Dienes (2008), an adjusted standard error was applied using the following equation: *SE* ∗ (1 + 20 / *df* ∗ *df*).

4 In order to match the pots/pans to the alcohol memory images, which had a greater variety of colours within each image, the selected exemplar within each image was colourised to another colour that was suitable for a pot or pan (e.g. switching stainless steel to a copper colour). Nine images were changed in this way.
