# Supplementary material for: Attentional capture by alcohol-related stimuli may be activated involuntarily by top-down search goals
Source: Psychopharmacology (Berl). 2018 Apr 25;235(7):2087–99. doi: 10.1007/s00213-018-4906-8 (PMC6015597; doi:10.1007/s00213-018-4906-8)
Supplement: Supplementary file 1 — (DOCX 15 kb) [file 213_2018_4906_MOESM1_ESM.docx]

**Attentional capture by alcohol related stimuli may be activated involuntarily by top-down search goals**

Chris R.H. Brown+, Theodora Duka+ & Sophie Forster+*

+ School of Psychology, University of Sussex, UK

*Corresponding author

School of Psychology, University of Sussex, Falmer, BN19QH, United Kingdom

Email: [s.forster@sussex.ac.uk](mailto:s.forster@sussex.ac.uk)

**Online materials 1**

**Individual study analysis**

**Experiment 1a.** Using *A’* as a dependent measure, a 2×3 repeated measures ANOVA, with Target (Pots/pans, Alcohol) as one factor and Distractors (Pots/pans, Alcohol, Shoes) as the other factor. This revealed a significant main effect of target, *F*(1,11) = 17.42, *p* = .002, *ƞ^2^_p_* = .61, with alcohol stimuli being detected significant more than the pots and pans stimuli. There was also a main effect of distractor, *F*(2,22) = 5.22, *p* = .014, *ƞ^2^_p_* = .32. This effect appeared to be primarily driven by the significant interaction term, *F*(2,22) = 5.79, *p* = .019 (Huynh-Feldt corrected), *ƞ^2^_p_* = .34, whereby alcohol distractors reduced detection sensitivity considerably more in the alcohol search condition.

To explore this pattern of results we ran Bayesian pairwise comparisons and two tailed t-tests between the shoe distractors, which were always goal incongruent, and the alcohol and pot distractors in both search goal conditions. This revealed that there was evidence which favoured the null for pot distractors when they were incongruent with the current alcohol search goal, *t*(11) = .65, *p* = .532, B_H[0, .10]_ = .20. This was also found for alcohol distractors when they were incongruent with the current pot search goal, *t*(11) = .76, *p* = .461, B_H[0, .10]_ = .13. When the pot distractor was congruent with the pot search goal, there was evidence of a null effect, *t*(11) = 1.17, *p* = .265, B_H[0, .10]_ = .49, though the difference in the congruent condition suggested an insensitive effect (Bayes factor > .33). However, when the alcohol distractor was congruent with the alcohol search goal, evidence favoured the experimental hypothesis, *t*(11) = 3.82, *p* = .003, B_H[0, .10]_ = 22.91. Thus, the interaction was driven purely by goal-driven capture by alcohol distractors.

**Experiment 1b.** The same analysis for Experiment 1b revealed a significant effect of target, *F*(1,15) = 9.76, *p* = .007, *ƞ^2^_p_* = .39, with participants correctly detecting the alcohol target more than the pots and pans targets. There was also a significant effect of distractor on detection sensitivity, *F*(2,30) = 11.09, *p* = .001 (Huynh-Feldt corrected), *ƞ^2^_p_* = .43, importantly this distractor effect was qualified by a significant interaction between target and distractor, *F*(2,30) = 12.47, *p* = .001 (Huynh-Feldt corrected), *ƞ^2^_p_* = .45, with the alcohol distractor and pot distractor being having reduced distractor sensitivity relative to the shoe distractor only when they were congruent with the current search goal on that block.

The Bayesian pairwise comparisons and two tailed t-tests revealed that, as in Experiment 1a, there was substantial evidence of a difference between the shoe distractor and the alcohol distractor in the alcohol search condition, *t*(15) = 3.45, *p* = .004, B_H[0, .10]_ = 120.77, however, evidence favoured the null hypothesis of no effect when the alcohol distractor was incongruent with the pot search goal, *t*(15) = .48, *p* = .638, B_H[0, .10]_ = .40. Unlike Experiment 1a, there was substantial evidence that pot distractors reduced detection sensitivity relative to shoe distractors in the pot search condition, *t*(15) = 2.84, *p* = .012, B_H[0,.10]_ = 12.40. Importantly, evidence favoured a null effect when the pot distractor was incongruent with the current alcohol search goal, *t*(15) = 1.10, *p* = .287, B_H[0, .10]_ = .30, suggesting that there was no reduction in *A’.*

**Experiment 1c.** As in previous investigations *A’* detection sensitivity was the dependent variable. This measure was compared across conditions in a 2×3 repeated measures ANOVA, with Target (Shoe, Alcohol) as one factor and Distractors (Shoes, Alcohol, Pots) as the other factor. This revealed a non-significant effect of target, *F*(1,59) = 1.34, *p* = .252, *ƞ^2^_p_* = .02, with no reliable difference between the detection sensitivity of the shoe and alcohol targets. There was, however, a significant difference between the three distractors, *F*(1,118) = 26.59, *p* < .001, *ƞ^2^_p_* = .31, with both alcohol and shoe distractors resulting in lower detection sensitivity than the pot distractor. Importantly, this was qualified by a significant two-way interaction, *F*(2,118) = 25.12, *p* < .001, *ƞ^2^_p_* = .3, with both alcohol and shoe distractors resulting in lower detection sensitivity when congruent with the target, relative to when it was incongruent with the current goal.

To explore this further, we conducted Bayesian pairwise comparisons between the goal relevant distractors and the goal irrelevant distractor across both search goal conditions. This revealed that there was no difference between *A’* when the distractor was an alcoholic stimulus compared to when it was a goal irrelevant pot stimulus, but only when it was incongruent with the current shoe search goal, *t*(59) = 1.27, *p* = .209, B_H[0,.10]_ = .24. Conversely, when participants were searching for alcohol, there was substantial evidence of a decrement in *A’* when the distractor was an alcohol stimulus compared to the goal irrelevant pot stimulus, *t*(59) = 5.94, *p* < .001, B_H[0,.10]_ = 9334567. A similar pattern was also observed with the shoe distractors: Evidence favoured a null effect when comparing *A’* between the shoe distractors and the pot distractors when participants were not currently searching for shoes, *t*(59) = .10, *p* = .917, B_H[0, .10]_ = .05. However, when participants were searching for shoe targets, shoe distractors resulted in substantial evidence of a decrement in *A’* relative to pot distractors, *t*(59) = 4.60, *p* < .001, B_H[0, .10]_ = 9043.12.
