# Supplementary material for: Attentional capture by alcohol-related stimuli may be activated involuntarily by top-down search goals
Source: Psychopharmacology (Berl). 2018 Apr 25;235(7):2087–99. doi: 10.1007/s00213-018-4906-8 (PMC6015597; doi:10.1007/s00213-018-4906-8)
Supplement: Supplementary file 2 — (DOCX 21.6 kb) [file 213_2018_4906_MOESM2_ESM.docx]

**Attentional capture by alcohol related stimuli may be activated involuntarily by top-down search goals**

Chris R.H. Brown+, Theodora Duka+ & Sophie Forster+*

+ School of Psychology, University of Sussex, UK

*Corresponding author

School of Psychology, University of Sussex, Falmer, BN19QH, United Kingdom

Email: [s.forster@sussex.ac.uk](mailto:s.forster@sussex.ac.uk)

**Online materials 2**

**Exploratory analyses across alcohol relevant measures**

**Comparisons across alcohol dependence groups.**  In order to assess the influence of current alcohol dependence we divided participants in Experiment 1c (the experiment with the largest sample; *n* = 60) into two groups based on their AUDIT score. This produced one group of 14 individuals who were at high risk of alcohol dependence (AUDIT > 15) and a group of 14 individuals who were low risk of alcohol dependence (AUDIT < 8). The cut-offs selected were based on guidelines for clinical intervention for alcohol use disorders (cf. Barbor et al. 2001).

We repeated the 2×3 repeated measures ANOVA in the main analysis for Experiment 1c, but with the inclusion of alcohol dependence grouping as a between-subjects factor. The ANOVA revealed that, as before, there was no significant difference between shoe and alcohol search goals, *F*(1,26) = .44, *p* = .511, *ƞ^2^_p_* = .02. As before, there was a significant difference between the different distractor types, *F*(2,52) = 14.46, *p* < .001, *ƞ^2^_p_* = .36 (Greenhouse-Geisser corrected). This was qualified with a significant interaction between search goal and distractor type, *F*(2,52) = .03, *p* = .897 (Greenhouse-Geisser corrected).

Importantly, the inclusion of the two levels of problem drinking (low/high risk of dependence) in the analysis revealed no significant interaction effects. Neither search goal, *F*(1,26) < .01, *p* = .965, *ƞ^2^_p_* < .01, nor distractor type, *F*(2,52) = 13.44, *p* = .743, *ƞ^2^_p_* = .01, significantly interacted with the between subjects factors. Importantly, the three-way interaction was also non-significant, *F*(2,52) = .03, *p* = .888, *ƞ^2^_p_* < .01 (all interactions were Greenhouse-Geisser corrected), therefore showing that individuals who were at high risk of alcohol dependence were no more, or less, distracted by alcohol than low risk drinkers in either search condition.

To determine the strength of evidence favouring distraction by alcohol stimuli within both groups, we conducted Bayesian pairwise comparisons between the alcohol distractors and the completely irrelevant non-alcohol distractors (pots/pans) within the goal-driven distraction condition (alcohol search), as well as the stimulus-driven distraction condition (shoe search). We did this for each group independently, thus yielding four pairwise comparisons. The prior for the Bayes factor was set at .10 for both groups, which was the same as previous Bayesian comparisons for the overall sample. Due to the smaller samples of this analysis (*n* < 30) we adjusted the standard error using the following equation: SE*(1 + 20/df*df) (cf. Dienes, 2008).

This revealed that both the low and high risk of dependence drinkers showed substantial evidence of a goal-driven distractor effect by alcohol, *t*(13) = 3.06, *p* = .009, B_H[0,.10]_ = 18.52; *t*(13) = 3.17, *p* = .007, B_H[0,.10]_ = 23.43, respectively. However, the comparison in the stimulus-driven condition for the high risk drinkers showed some slight evidence favouring distraction by alcohol stimuli, *t*(13) = 2.39, *p* = .033, B_H[0,.10]_ = 2.04. Whilst the low risk drinkers showed strong evidence favouring the null hypothesis for the same effect, *t*(13) = .33, *p* = .750, B_H[0, .10]_ = .17. It therefore appears that there may be some small evidence of a stimulus-driven effect in individuals who are at risk of alcohol dependence which warrants further investigation. Though this evidence favours IST, with high risk drinkers showing some evidence of a stimulus-driven effect, we note that this *post hoc* analysis would not survive corrections for multiple comparisons (α = .013), and that the evidence favouring the experimental effect was weak (Bayes factor < 3).

**Relationships between distraction and alcohol measures.** In order to explore whether there was any systematic variation across alcohol distractor effects depending on alcohol related measures, we computed Pearson’s correlation coefficients between alcohol related self-report measures and both goal-driven (alcohol search) and stimulus-driven (shoe search) alcohol distractor effects.

Bayes factors were computed to test whether the results favoured the null (i.e. no relationship) or the experimental hypothesis (i.e. a positive relationship between alcohol questionnaire measure and distractor effect). The prior for all correlational analysis was set as .27 as the upper-limit of the expected effect size based on the Fisher’s *Z* transformed effect size (*r* = .26) taken from a meta-analysis of the correlation between attentional bias to addictive substances and substance use (Rooke et al. 2008). This relatively small expected effect size was selected based on the relationship being taken from multiple types of experiment, and should therefore generalise to our own novel task.

The alcohol relevant self-report measures included the number of units drank per week and the binge score, derived from the Alcohol Use Questionnaire (AUQ; Mehrabian and Russell 1978), the Alcohol Use Disorders Identification Test (AUDIT; Sanders et al. 1993), and the positive high arousal subscale of the Anticipated Effects of Alcohol Scale (AEAS; Morean et al. 2012). All p-values showed a non-significant effect, and the Bayes factors revealed insensitive data, being neither lower than .33 nor higher than 3, and can only be described as a weak anecdotal relationships (see Table 1; Dienes 2008).

|  | Goal-driven alcohol distraction | | | Stimulus-driven alcohol distraction | | |
| --- | --- | --- | --- | --- | --- | --- |
|  | Pearson’s r | p value | Bayes factor | Pearson’s r | p value | Bayes factor |
| Units | -.10 | .44 | .27 | .15 | .245 | 1.32 |
| Binge score | .06 | .643 | .63 | .03 | .811 | .53 |
| AUDIT | .03 | .852 | .51 | .16 | .213 | 1.42 |
| AEAS – positive high arousal | -.05 | .698 | .34 | -.05 | .700 | .34 |

**Table 1.** Pearson’s r correlation coefficient, p values, and Bayes factors for the relationship between distractor effects when congruent or incongruent with the current search goal, and alcohol related self-report measures. These measures include the number of units drank per week and the binge score derived from the AUQ (Mehrabian and Russell 1978). The AUDIT (Sanders et al. 1993), and the positive high arousal subscale of the AEAS (Morean et al. 2012).

**References**

Dienes Z (2008) Understanding psychology as a science: An introduction to scientific and statistical inference Hampshire, England: Palgrave Macmillan Retrieved from [http://wwwlifescisussexacuk/home/Zoltan _Dienes/inference/](http://wwwlifescisussexacuk/home/Zoltan%20_Dienes/inference/)

Mehrabian A, Russell JA (1978) A questionnaire measure of habitual alcohol use. Psychol Rep 43:803-806.

Morean ME, Corbin WR, Treat TA (2012) The Anticipated Effects of Alcohol Scale: development and psychometric evaluation of a novel assessment tool for measuring alcohol expectancies. Psychol Ass 24:1008-1023.
